# Kinematics of the head and associated vertebral artery length changes during high-velocity, low-amplitude cervical spine manipulation

**DOI:** 10.1186/s12998-022-00438-0

**Published:** 2022-06-01

**Authors:** Lindsay M. Gorrell, Gregor Kuntze, Janet L. Ronsky, Ryan Carter, Bruce Symons, John J. Triano, Walter Herzog

**Affiliations:** 1 Human Performance Laboratory, Faculty of Kinesiology, University of Calgary, Calgary, Canada; 2 Integrative Spinal Research Group, Department of Chiropractic Medicine, University Hospital Balgrist and University of Zürich, Zurich, Switzerland; 3 Sports Injury Prevention Research Centre, Faculty of Kinesiology, University of Calgary, Calgary, Canada; 4 Mechanical and Manufacturing Engineering, Schulich School of Engineering, University of Calgary, Calgary, Canada; 5 Private practice, Calgary, Canada; 6 Canadian Memorial Chiropractic College, Toronto, Canada

**Keywords:** Cervical spine, Kinematics, Spinal manipulation, Spontaneous vertebral artery dissection, Strain

## Abstract

**Background:**

Cervical spine manipulation (CSM) is a frequently used treatment for neck pain. Despite its demonstrated efficacy, concerns regarding the potential of stretch damage to vertebral arteries (VA) during CSM remain. The purpose of this study was to quantify the angular displacements of the head relative to the sternum and the associated VA length changes during the thrust phase of CSM.

**Methods:**

Rotation and lateral flexion CSM procedures were delivered bilaterally from C1 to C7 to three male cadaveric donors (Jan 2016–Dec 2019). For each CSM the force–time profile was recorded using a thin, flexible pressure pad (100–200 Hz), to determine the timing of the thrust. Three dimensional displacements of the head relative to the sternum were recorded using an eight-camera motion analysis system (120–240 Hz) and angular displacements of the head relative to the sternum were computed in Matlab. Positive kinematic values indicate flexion, left lateral flexion, and left rotation. Ipsilateral refers to the same side as the clinician's contact and contralateral, the opposite. Length changes of the VA were recorded using eight piezoelectric ultrasound crystals (260–557 Hz), inserted along the entire vessel. VA length changes were calculated as D = (L_1 _− L_0_)/L_0_, where L_0_ = length of the whole VA (sum of segmental lengths) or the V3 segment at CSM thrust onset; L_1_ = whole VA or V3 length at peak force during the CSM thrust.

**Results:**

Irrespective of the type of CSM, the side or level of CSM application, angular displacements of the head and associated VA length changes during the thrust phase of CSM were small. VA length changes during the thrust phase were largest with ipsilateral rotation CSM (producing contralateral head rotation): [mean ± SD (range)] whole artery [1.3 ± 1.0 (− 0.4 to 3.3%)]; and V3 segment [2.6 ± 3.6 (− 0.4 to 11.6%)].

**Conclusions:**

Mean head angular displacements and VA length changes were small during CSM thrusts. Of the four different CSM measured, mean VA length changes were largest during rotation procedures. This suggests that if clinicians wish to limit VA length changes during the thrust phase of CSM, consideration should be given to the type of CSM used.

## Introduction

**Fig. 1:**
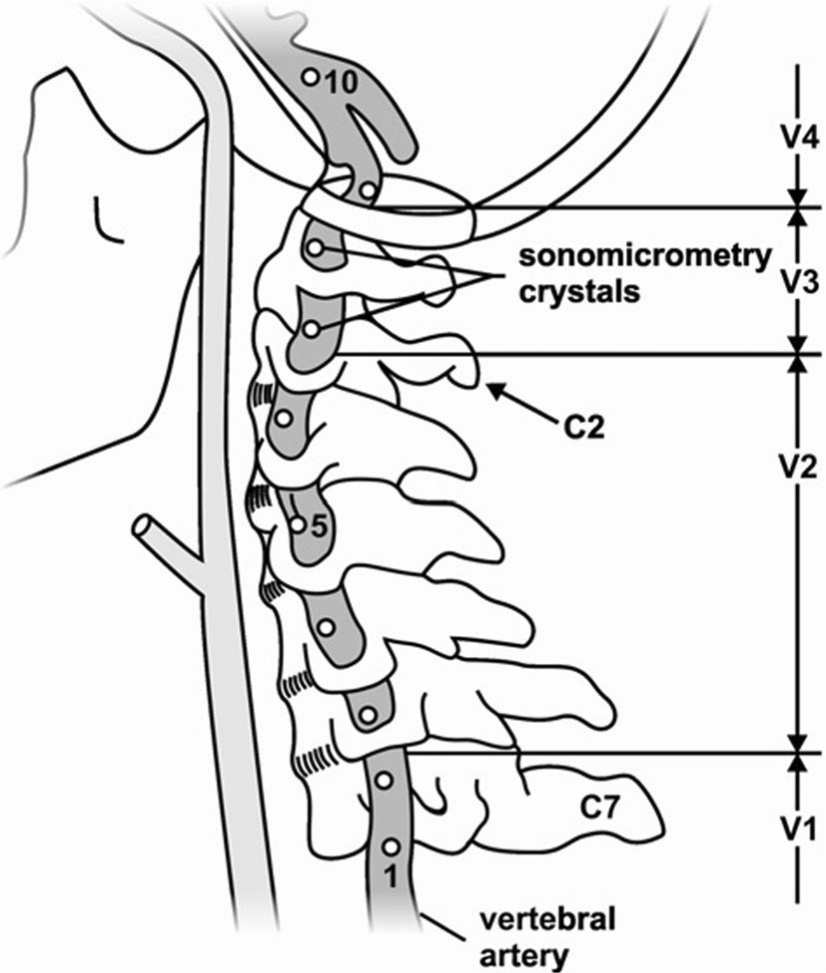
Schematic of ultrasound crystal placement. Adapted from Wuest et al. [30]. Legend: cervical vertebra (C); vertebral artery region (V); ultrasound crystals (numbers 1–8)

Neck pain is a common cause of musculoskeletal pain in the adult population, with annual global prevalence estimates in the range of 17–75% and costs in excess of US$ 8 billion/year in the United States alone [1–3]. Cervical spine manipulation (CSM) is a frequently used treatment modality for patients with neck pain [4, 5] and is recommended in many clinical practice guidelines [6–8]. Despite its demonstrated efficacy [9, 10], concerns remain surrounding the safety of CSM [11–14]. It has been suggested that head and neck extension and rotation during some CSM may stretch and damage the vertebral artery (VA) wall, leading to arterial dissection and stroke [12, 15, 16]. Such damage predominantly occurs in the V3 segment of the artery, which may be vulnerable with elongation (Fig. 1), highlighting the importance of investigating length changes in this segment during CSM [17–19].

One approach to investigate the relationship between movement and elongation of the VA during CSM is to quantify the kinematics of the head and associated VA length change. The kinematics of the head relative to the sternum during CSM have been investigated in both asymptomatic live [20–24] and cadaveric subjects [25, 26]. Despite the use of varying CSM techniques, the current literature reports that head angular displacements during CSM are small, especially for rotation movements [25], and they do not exceed the normal physiological range of motion [21, 23]. However, in an early study, angular head displacements were shown to approach the maximal active range of motion for the upper cervical spine at the pre-manipulative position [20]. A study by Piper et al. [26] remains the only investigation in which the kinematics of the head relative to the sternum and the associated VA length changes were measured simultaneously. However, in that study, head kinematics and VA length changes at peak CSM force occurrence were reported relative to the VA length and head/neck position in the neutral anatomical position [26]. Angular displacements and associated VA length changes during the thrust phase of CSM were not separately reported in that study and therefore remain unknown. Furthermore, it has been suggested that the force and amplitude of CSM are responsible for VA damage [15, 18, 27] and indeed, any ‘rapid jerking movement’ [28]. Since the thrust phase of CSM involves a rapid increase in the applied forces and (possibly) amplitudes, it is important to quantify angular displacements of the head/neck and VA length during this phase of the procedure.

Including the Piper et al. investigation, four studies have quantified the elongation response of the VA to CSM and passive ranges of motion [26, 29–31]. In these studies, arterial length changes were reported for specific regions [26, 29] or, along the entire course of the artery [30, 31] following CSM delivered to a maximum of three vertebral levels. Collectively, it was found that from a neutral anatomical head and neck position, movements involving contralateral (opposite to the side of VA instrumentation) head rotation resulted in the largest VA length changes in the V3 segment during both CSM (range − 15 to 18%) and passive ranges of motion (0–38%) [26, 29–31]. From a neutral head and neck position, VA length changes measured during CSM were typically lower than those measured during passive range of motion testing and did not approach published failure length changes (also measured as strains from a neutral head and neck position [153–162%]) [29]. Further, on average, the VA must elongate about 12% prior to mechanical failure when measured from a standardized neutral anatomical position [31].

Despite these reports, the kinematics of the head relative to the sternum and associated VA length changes during the thrust phase of CSM delivered systematically to each level of the cervical spine (C1–C7) have not been investigated. Therefore, it is unknown if VA length changes differ during the thrust phase with CSM applied to different levels of the cervical spine. Furthermore, in previous studies [26, 29–31], total length changes of VA from a neutral anatomical reference position were reported. The length changes of the VA for these situations might be thought of as the length change from the reference position to the pre-manipulative position (setup phase) plus the length change of the VA during the thrust phase of the CSM. However, it is unknown how much each phase contributes to the total VA length changes during CSM.

Therefore, the purpose of this study was to systematically quantify the angular displacements of the head relative to the sternum and the associated VA length changes during the thrust phase of two types of CSM (rotation and lateral flexion), applied bilaterally, to each level of the cervical spine (C1–C7). It was hypothesized that there would be no differences in VA length changes (whole artery or V3 segment) during CSM applied to the different vertebral levels (e.g. C1 vs. C2).

## Methods

### Donor recruitment and preparation

Three male cadaveric donors were secured through the University of Calgary’s Body Donation Program (January 2016—December 2019). The study was approved by the Conjoint Health Research Ethics Board (REB16-0296) of the University of Calgary. Visual inspection revealed no substantial anatomic variations in the origin, course or appearance of the VA. Minor osteophytes were present in the cervical spine of all donors; however, this did not affect the passive ranges of motion of the neck assessed qualitatively prior to dissection. Blunt dissection of the anterior cervical region was performed by a trained anatomist (~ 10 years’ experience) to expose the VA. All efforts were made to ensure that the minimum amount of tissue was removed to approximate, as closely as possible, the contributions of soft tissues of the neck to movement. A single VA was instrumented with 2 mm piezoelectric ultrasound crystals (Sonometrics Corporation, London, Canada). Eight crystals were inserted into the lateral aspect of the artery’s lumen along its entire length and secured to the arterial wall using three non-collinear sutures (Fig. 1). Great care was taken to maintain consistency in crystal spacing (10–30 mm) and location across all donors. Crystals 1–8 were inserted as follows: (1) at the mid-point between the subclavian artery and the C6 transverse foramen; (2–5) at the mid-point between adjacent transverse foramen of C6 to C2; (6, 7) adjacent to the C2 and C1 transverse foramen respectively and; (8) distal to the C1 transverse foramen.

There were 2 instances when it was not possible to follow this exact pattern due to normal anatomical variations between donors. Anatomical variations included limited space between adjacent transverse foramen prevented placement of crystals and enlarged cervical nerve roots exiting the neural foramen [32]. Following crystal placement, single 3 mm stainless steel surgical bone pins (IMEX Veterinary Inc, Longview, TX, USA) were introduced into the skull and sternum. Dental cement (Bosworth Company, Skokie, IL, USA) was used to secure the pins with a curing time of at least 10 h. During this time, all exposed tissues were covered in gauze soaked in a physiological saline solution. Where possible, the duration of dissection and instrumentation (~ 16 to 24 h) was minimized and when no active work was occurring, the cadaver was stored at 4 °C to reduce tissue deterioration. Prior to data collection, prefabricated triads consisting of three non-collinear, 10 mm diameter retroreflective marker spheres were firmly affixed to each bone pin using quick-setting steel reinforced epoxy (JB Kwik Weld, Sulphur Springs, TX, USA).

### Data collection

Three clinicians (clinical experience 7–20 years) performed all CSM. For each donor, data were collected from two clinicians, thus different individuals contributed to the data. The in-situ head position was taken as the arbitrary position that the skull assumed when positioned on the gurney, and was not controlled within or between donors. This position was determined by the bony anatomy of the head and neck alone and was not controlled. The order of manipulation delivery was random and established using the randomized number generator function in Matlab (vR2019b; Mathworks, USA). Clinicians delivered a single supine, Diversified style (manual, high-velocity low-amplitude) CSM (rotation and lateral flexion) to each cervical vertebra (C1 to C7) on both sides of the neck [33]. For all procedures, the articular column of the involved vertebra was targeted through the intact posterior tissues by the antero-lateral aspect of the proximal phalanx of the clinician’s second digit. The pre-manipulative position involved head and neck flexion, ipsilateral (same side as the clinician's contact) lateral flexion, and contralateral (opposite side to the clinician's contact) rotation. The pre-manipulative position was defined as the position of the head and neck at the instant of the rapid increase in manipulative force following the relatively steady pre-manipulative force and indicated the onset of the manipulative thrust. From the pre-manipulative position, a rapid, controlled low-amplitude thrust was applied in an intended posterior-anterior (rotation) or medial and slightly inferior (lateral flexion) direction [33].

**Fig. 2:**
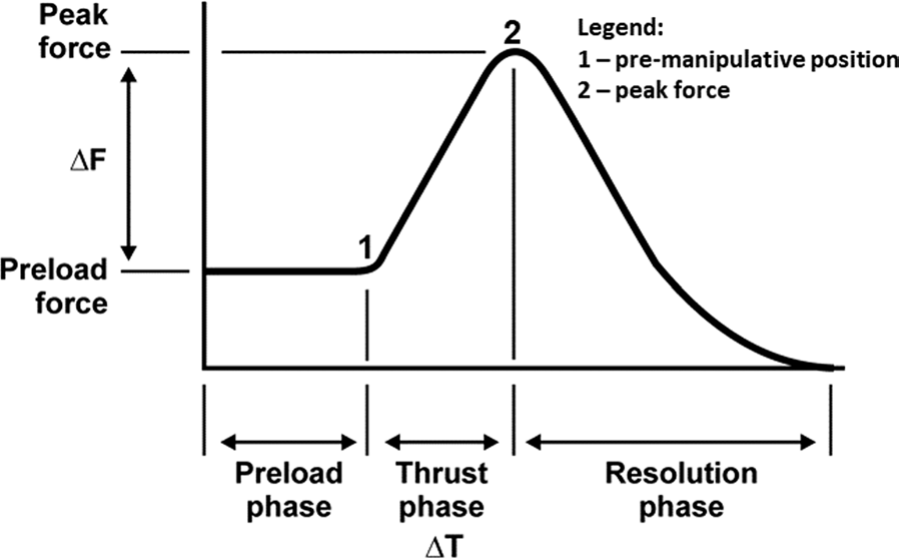
Typical force–time profile for spinal manipulation. Legend: change in (Δ); force (F), time (T)

During each trial, VA length changes in a single VA were captured using a SonoSoft system (Sonometrics Corporation, London, ON, Canada; 260–557 Hz) with a spatial resolution of 16 µm [29]. Prior to, and as necessary throughout data collection, arteries were perfused with ultrasonographic gel to approximate their *in-vivo* shape and to promote ultrasound signal transmission. For each CSM, the force–time profile was recorded using a thin, flexible pressure pad (Pedar-X, Novel, Munich, Germany; ~ 20 cm × 10 cm × 0.2 cm, 100–200 Hz), enabling identification of the time of the pre-manipulative position (thrust onset) and peak force (end of the thrust phase) (Fig. 2). A sampling rate of 100 Hz was used in the first data collection. This was increased to 200 Hz for all subsequent data collection. A sampling rate of 100 Hz is adequate to quantify high-velocity, low-amplitude SM thrusts [34]. The pad was placed securely between the clinician’s contact and the donor’s neck [35]. Three-dimensional (3D) angular displacements of the head relative to the sternum were recorded using an eight-camera optical motion capture system (Motion Analysis, Santa Rosa, CA, USA; 120–240 Hz video, 2400 Hz analogue). A sampling rate of 120 Hz was used in the first data collection. This was increased to 240 Hz for all subsequent data collection. All data were time synchronized using a square wave 5 V electrical pulse at the beginning of each trial. The rising edge of the synchronization pulse was identified in a Matlab script (vR2019b; Mathworks, USA) and designated as time zero across systems. Thereafter, data frames for each system were converted to time in seconds based on the respective sampling frequencies. This approach enabled data extraction across systems at common event timings. Following data collection, Computed Tomography (CT) images of the donor skull to the level of the thoracic spine were acquired (Revolution GSI, GE Healthcare, Chicago, IL, USA).

### Data analysis

VA lengths acquired with the SonoSoft system were exported to Excel (Microsoft Office 365, Redmond, WA, USA). Intersegmental elongations were summed to give: (i) overall VA length change along the entire artery and; (ii) VA length change in the V3 segment (Fig. 1). VA length change (D) was calculated as:

$${\text{D}} = ({\text{L}}_{1} - {\text{L}}_{0} ){\text{/L}}_{0} ,$$

where L_0_ was the instantaneous length of the artery at the pre-manipulative position, and L_1_ the instantaneous length of the vessel at the time of peak force during the manipulative thrust.

**Fig. 3:**
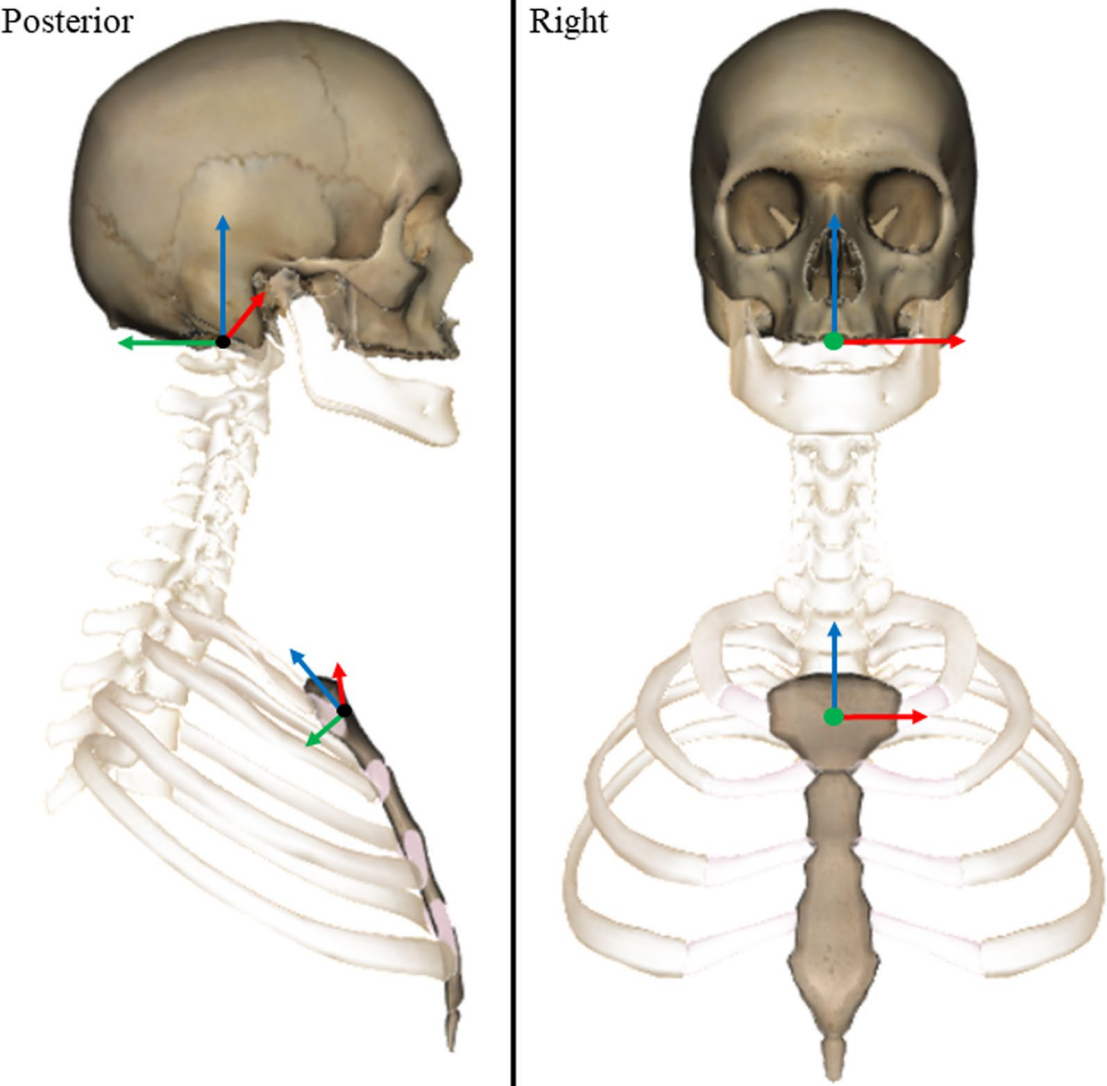
Origins and orthonormal local co-ordinate systems for the skull and sternum: X axis—segmental flexion/extension (red); Y axis—segmental lateral flexion (green) and; Z axis—axial rotation (blue)

Reflective marker positions were tracked using Cortex software (v5.02, Motion Analysis, CA, USA) and filtered using an 8 Hz, low pass, 4th order zero-lag Butterworth filter in Matlab [36]. Orthonormal coordinate systems were defined for the skull and sternum in Matlab using donor-specific 3D bone models created using manual segmentation from the CT images (Mimics, v21, Materialise NV, Belgium). The origin of the skull was located in the centre of the foramen magnum. The origin was defined using the mean coordinates of the mid-points of the lines connecting (1) the left and right inferior lateral, and (2) the anterior and posterior inferior aspects of foramen magnum (Fig. 3). The medial–lateral axis was defined in the direction of the inferior lateral aspects of the foramen magnum. The superior-inferior axis was defined using the cross product of the vectors of the medial–lateral and intermediate anterior–posterior axis (i.e. inferior anterior and posterior aspects of foramen magnum). The final anterior–posterior axis was defined as the cross product of the vectors representing the medial–lateral and superior-inferior axes. The origin of the sternum was defined as the mean coordinates of the mid-points of the lines connecting (1) the left and right lateral inferior aspects of the articular facets for the clavicles, and (2) the most superior and inferior aspects of the midline of the sternum. The medial–lateral axis was defined in the direction of the lateral aspects of the sternum. The anterior–posterior axis was defined using the cross product of the vectors of the medial–lateral and intermediate superior-inferior axis (i.e. superior and inferior aspects of the midline). The final superior-inferior axis was defined as the cross product of the vectors representing the medial–lateral and anterior–posterior axes. The coronal axis (X), was defined as positive to the left, the sagittal axis (Y), positive posteriorly, and the transverse axis (Z), positive superiorly (Fig. 3). Change in head angular displacements relative to the three axes of the sternum LCS were calculated from the time of onset to the time of peak force occurrence during the CSM thrust.

### Statistical analysis

Descriptive statistics [mean ± standard deviation (SD), (range)] were used to report the angular displacement of the head relative to the sternum and the VA length changes (whole vessel and V3 segment) during CSM. Differences in VA length changes between adjacent cervical vertebra (*i.e.* C1 compared to C2 etc.) were evaluated using the Wilcoxson Signed Rank Testing Exact method (SPSS, version 27, IBM, USA). Statistical significance was set at *p* < 0.05.

## Results

One hundred and sixty-eight CSM were delivered to three male cadaveric donors (88 ± 6 years old; Table 1) in this study, with 165 being used for analysis. There were no significant differences in VA length changes (whole artery or V3 segment) during CSM applied to the different vertebral levels (e.g. C1 vs. C2) (Tables 2, 3, 4). Therefore, the descriptive statistics were calculated by pooling data from all CSM; *i.e.* thrusts delivered to each level of the cervical spine (C1 to C7) and on both sides of the neck for all donors and all clinicians (Table 5).

**Table 1:** Donor demographics

	Sex	Age (yrs)	Height (cm)	Weight (kg)	Time since death until testing (hr)	Reason for death	Pre-existing conditions
	M	82	178	71	65	Dementia	Spinal stenosis, Diabetes Mellitus II
	M	94	168	70	144	Congestive cardiac failure	Unknown
	M	87	170	57	144	Obstructive pneumonia	Metastatic lung and colon cancer, chronic obstructive pulmonary disease, benign prostatic hypertrophy
Mean ± SD		88 ± 6	172 ± 5	66 ± 8	118 ± 46		

M, male; yrs, years; cm, centimeters; kg, kilograms; hr, hours; SD, standard deviation

**Table 2:** Angular displacement (degrees) of the head relative to the sternum and VA length change (%) combining data from all donors and clinicians during ipsilateral CSM thrusts

		Rotation					Lateral flexion				
		X	Y	Z	Whole	V3	X	Y	Z	Whole	V3
C1	Mean	2.5	8.1	− 11.5	1.1	3.7	− 3.0	4.9	− 4.4	0.9	2.6
	SD	2.6	2.8	3.8	0.9	4.4	1.7	4.3	7.8	1.5	4.3
	Minimum	0.2	5.9	− 15.0	0.0	− 0.3	− 5.3	0.4	− 20.0	− 1.1	0.0
	Maximum	6.5	12.5	− 5.8	2.3	10.0	− 0.9	10.7	0.7	3.2	10.4
C2	Mean	2.1	10.0	− 12.6	1.6	− 0.1	− 5.3	5.6	− 6.4	1.1	2.3
	SD	4.4	3.5	4.5	1.3	5.3	4.2	3.2	8.9	0.8	3.6
	Minimum	− 3.8	− 5.0	− 16.1	0.1	− 9.3	− 13.0	1.0	− 19.3	0.2	− 0.3
	Maximum	7.6	13.9	− 3.7	3.2	7.1	− 1.6	9.8	7.9	2.1	8.9
C3	Mean	− 0.3	7.2	− 11.0	1.9	4.5	− 3.6	4.7	− 4.1	0.7	1.7
	SD	2.1	2.3	2.6	0.7	5.4	2.2	3.2	3.0	0.5	2.6
	Minimum	− 1.9	4.4	− 13.7	0.8	0.0	− 7.0	1.1	− 8.9	0.2	0.0
	Maximum	2.8	10.1	− 7.5	2.4	10.8	− 1.4	9.4	− 0.9	1.3	6.7
C4	Mean	1.5	8.9	− 9.8	1.4	2.8	− 3.9	6.3	− 5.1	0.6	2.1
	SD	2.6	4.5	3.7	1.0	4.0	3.1	2.5	5.1	1.0	2.6
	Minimum	− 2.8	2.5	− 14.5	0.2	0.0	− 9.1	2.2	− 10.7	− 0.6	0.0
	Maximum	5.0	13.2	− 5.6	2.7	8.6	− 1.7	9.1	2.0	2.1	5.6
C5	Mean	1.4	9.6	− 10.7	1.8	2.5	− 3.9	5.7	− 2.1	1.2	2.5
	SD	4.7	2.7	3.1	1.2	3.1	3.0	3.8	4.2	1.0	2.6
	Minimum	− 6.1	5.7	− 15.5	− 0.1	0.0	− 7.6	1.3	− 6.0	0.2	0.0
	Maximum	5.1	12.6	− 7.4	3.1	6.6	− 0.9	12.6	5.5	2.6	6.4
C6	Mean	1.3	8.9	− 8.0	1.0	1.8	− 4.2	4.6	− 5.4	1.3	2.4
	SD	3.2	3.0	2.6	1.2	3.7	3.2	2.0	6.3	0.7	2.4
	Minimum	− 3.9	5.0	− 12.0	− 0.4	− 0.4	− 9.4	1.5	− 10.6	0.7	0.0
	Maximum	5.5	14.1	− 4.6	3.1	11.6	− 1.1	6.5	5.1	2.1	5.8
C7	Mean	− 0.8	8.7	− 9.5	1.3	2.0	− 3.5	5.4	− 3.3	1.2	2.0
	SD	4.3	2.6	3.0	1.1	2.8	3.5	2.6	4.1	1.0	3.6
	Minimum	− 6.6	6.0	− 15.3	0.3	− 0.1	− 8.8	2.3	− 7.7	− 0.3	− 1.1
	Maximum	3.9	12.3	− 7.0	3.3	6.8	0.8	7.9	2.5	2.3	7.1

Ipsilateral manipulations involve contralateral head rotation; positive kinematic values indicate flexion, left lateral flexion and left rotation; positive VA length changes indicate elongation of the vessel

X, coronal axis; Y, sagittal axis; Z, transverse axis; SD, standard deviation; whole, whole VA; V3, V3 segment of VA

**Table 3:** Angular displacement (degrees) of the head relative to the sternum and VA length change (%) combining data from all donors and clinicians during contralateral CSM thrusts

		Rotation					Lateral flexion				
		X	Y	Z	Whole	V3	X	Y	Z	Whole	V3
C1	Mean	1.0	− 8.8	9.7	0.9	1.8	− 2.0	− 5.9	0.1	0.8	1.8
	SD	3.4	2.0	3.3	0.8	1.4	1.6	3.0	4.1	1.2	1.8
	Minimum	− 2.9	− 11.8	6.2	0.2	0.0	− 5.0	− 10.1	− 3.9	− 1.3	0.0
	Maximum	5.2	− 6.5	14.6	2.2	3.1	− 0.5	− 2.8	7.6	1.9	3.7
C2	Mean	0.9	− 7.0	9.7	0.6	0.9	− 2.3	− 5.6	− 0.3	1.6	2.0
	SD	2.8	3.0	3.2	0.4	1.0	3.3	5.7	2.2	1.2	2.6
	Minimum	− 2.6	− 10.3	5.4	0.2	0.0	− 7.3	− 11.5	− 4.0	0.1	0.0
	Maximum	4.2	− 2.6	13.6	1.1	2.2	1.8	4.4	2.3	3.6	6.0
C3	Mean	0.5	− 9.1	8.3	1.4	2.2	− 3.8	− 7.4	1.6	1.4	1.1
	SD	4.5	5.7	2.7	0.7	2.7	2.9	4.2	4.3	2.0	1.9
	Minimum	− 3.6	− 19.3	3.9	0.2	0.0	− 7.1	− 15.1	− 3.1	− 1.4	− 1.3
	Maximum	9.1	− 3.7	11.0	2.3	5.8	− 0.3	− 4.3	8.8	4.2	4.2
C4	Mean	0.1	− 8.2	7.4	1.0	1.3	− 4.3	− 6.0	1.8	1.8	0.7
	SD	2.0	3.9	2.5	0.7	1.8	4.5	2.3	6.8	1.7	2.1
	Minimum	− 2.5	− 14.5	5.1	− 0.3	− 0.7	− 10.1	− 10.1	− 7.8	0.0	− 1.3
	Maximum	3.5	− 3.8	11.6	1.6	3.5	1.0	− 3.6	12.9	4.4	3.9
C5	Mean	− 1.3	− 5.4	4.5	1.1	1.0	− 3.7	− 6.3	3.6	0.9	1.8
	SD	1.0	2.6	1.9	0.6	1.3	1.7	2.6	6.1	1.7	2.4
	Minimum	− 3.2	− 8.5	2.2	0.3	0.0	− 6.6	− 9.0	− 2.8	− 2.0	− 0.2
	Maximum	− 0.2	− 1.9	7.0	1.8	3.3	− 1.8	− 3.2	11.9	2.5	5.8
C6	Mean	− 0.7	− 8.3	7.5	1.2	1.4	− 4.6	− 5.2	0.8	1.5	1.7
	SD	3.5	1.6	2.6	0.2	1.5	2.3	3.2	6.4	0.7	2.0
	Minimum	− 7.7	− 10.9	3.3	1.0	0.0	− 6.1	− 8.8	− 4.9	0.6	0.0
	Maximum	1.7	− 6.3	10.4	1.5	4.0	− 1.2	− 1.3	9.9	2.3	3.9
C7	Mean	− 0.3	− 8.4	6.1	0.9	1.3	− 2.8	− 5.7	0.7	0.7	0.5
	SD	2.7	2.4	3.1	0.5	2.4	3.1	1.5	5.8	0.7	1.1
	Minimum	− 4.3	− 12.3	2.7	0.2	− 1.1	− 6.7	− 7.9	− 6.5	0.0	− 0.8
	Maximum	2.8	− 6.3	10.4	1.6	4.4	1.4	− 3.7	10.6	1.9	2.3

Contralateral manipulations involve ipsilateral head rotation; positive kinematic values indicate flexion, left lateral flexion and left rotation; positive VA length changes indicate elongation of the vessel

X, coronal axis; Y, sagittal axis; Z, transverse axis; SD, standard deviation; whole, whole VA; V3, V3 segment of VA

**Table 4:** Differences in VA length change between adjacent cervical spine levels during CSM thrusts

	VA length change during thrust between adjacent cervical spine levels			
	Rotation		Lateral flexion	
	Whole (*p* value)	V3 (*p* value)	Whole (*p* value)	V3 (*p* value)
C1–C2	0.850	0.055	0.339	0.945
C2–C3	0.375	0.232	0.301	0.547
C3–C4	0.322	0.846	0.520	0.910
C4–C5	0.365	0.910	0.898	0.203
C5–C6	0.638	> 0.999	0.164	0.641
C6–C7	0.898	0.846	0.301	0.641
C1–C7	0.831	0.148	0.791	0.109

whole, whole VA; V3, V3 segment of VA

Statistical significance (*p* < 0.05) was not achieved for any comparison

**Table 5:** Angular displacement (degrees) of the head relative to the sternum and VA length change (%) combining data from all cervical spine levels (C1 to C7), donors and clinicians during CSM thrusts

	Ipsilateral cervical spine manipulation										Contralateral cervical spine manipulation									
	Rotation					Lateral flexion					Rotation					Lateral flexion				
	X	Y	Z	Whole	V3	X	Y	Z	Whole	V3	X	Y	Z	Whole	V3	X	Y	Z	Whole	V3
Mean	1.2	8.8	− 10.2	1.3	2.6	− 3.9	5.3	− 4.4	1.0	2.2	0.0	− 7.9	7.7	1.0	1.4	− 3.3	− 6.1	1.2	1.2	1.4
SD	3.4	3.0	3.5	1.0	3.6	2.9	3.0	5.7	0.9	3.0	2.9	3.3	3.1	0.6	1.7	2.9	3.2	5.0	1.4	2.0
Minimum	− 6.6	2.5	− 16.1	− 0.4	− 0.4	− 13.0	0.4	− 20.0	− 1.1	− 1.1	− 7.7	− 19.3	2.2	− 0.3	− 1.1	− 10.1	− 15.1	− 7.8	− 2.0	− 1.3
Maximum	7.6	14.1	− 3.7	3.3	11.6	0.8	12.6	7.9	3.2	10.4	9.1	− 1.9	14.6	2.3	5.8	1.8	4.4	12.9	4.4	6.0

Ipsilateral manipulations involve contralateral head rotation; positive kinematic values indicate flexion, left lateral flexion and left rotation; positive VA length changes indicate elongation of the vessel

X, coronal axis; Y, sagittal axis; Z, transverse axis; SD, standard deviation; whole, whole VA; V3, V3 segment of VA

Irrespective of the type of CSM, the side or level of CSM application, angular displacements of the head and associated VA length changes during the thrust phase of CSM were small (Tables 2, 3, 5). Furthermore, visual inspection of the data revealed that there was considerable variability in the length changes measured in the whole artery and V3 segment across different donors and clinicians (Tables 2, 3, 5).

## Discussion

The purpose of this study was to quantify the angular displacements of the head relative to the sternum and the associated VA length changes during the thrust phase of two types of CSM (rotation and lateral flexion), applied bilaterally, to each level of the cervical spine (C1 to C7). The primary result of this study was that irrespective of the type of CSM, the side or level of CSM application, angular displacements of the head and associated VA length changes during the thrust phase of CSM were small (Tables 2, 3, 5) compared to those occurring at peak CSM force occurrence in the only other study to measure these two parameters simultaneously [26]. Additionally, visual inspection of the data revealed that there was considerable variability in the length changes measured in the whole artery and V3 segment across different donors and clinicians (Tables 2, 3, 5).

The current results report similar amounts of flexion, lateral flexion and axial rotation during the manipulative thrust as reported previously, despite methodological differences in the CSM technique used and vertebral level contacted [20, 22–25, 37]. Additionally, there did not appear to be a relationship between the vertebra to which the thrust was applied and the change in angular displacement of the head relative to the sternum during the thrust [21, 37]. Likewise, there were no significant differences in VA length changes when CSM thrusts were applied to the different vertebral levels (Table 4).

However, as the angular displacement of the head relative to the sternum was quantified, and VA length changes were measured for the entire VA and the V3 segment, it is possible that there are differences in the segmental motion and intersegmental VA length changes between adjacent cervical vertebrae. Supporting this argument for differences in segmental vs. global kinematics, it has been reported that angular displacements of individual cervical vertebrae in sagittal plane flexion/extension of the head may be greater at intermediate head flexion/extension angles than at maximal head flexion/extension angles [38]. Additionally, VA length changes were observed in opposite directions in adjacent motion segments i.e. elongation at the C1/2 level and shortening at the C2/3 level, consistent with one previous report [30].

Another consideration is that despite considerable variability, there was, on average, an elongation of the V3 segment – irrespective of the vertebra targeted by the CSM (Tables 2, 3, 5). This finding may be important as VA dissections occur more frequently in the V3 segment than in other areas of the VA [17–19]. However, it should be noted that even though the V3 segment usually elongated during CSM, there were instances when a shortening of the V3 segment was observed (maximum of − 9.3%) (Table 2). This highlights possible biomechanical differences in VA response between individuals receiving CSM. Visual inspection of the data did not reveal any patterns regarding VA response between donors or practitioners, thus it is unknown exactly what causes this variability but it is possible that anatomic differences in the course of the vessel (thus reducing the length of the V3 segment) [18] and/or variable CSM thrust delivery by different practitioners [39] may be important factors. Variability in the length changes associated with CSM has been reported previously [26, 29–31]. However, there does not appear to be a qualitative difference in the variability of VA length changes between the lateral flexion and rotation CSM delivered in this study.

### Limitations

This study involved cadaveric donors where dissection artifacts were unavoidable. While every effort was made to minimize alteration of tissues, dissection artifacts may have contributed to the mechanics of CSM and head motion that may not directly generalize to the clinical setting. Specifically, there could be differences in load transfer from the practitioner to the cadaver compared to from the practitioner to a live patient, due to the removal of soft tissues which could result in increased magnitudes of head displacements during CSM delivered to cadaveric donors. Thus, it is possible that with the removal of soft tissues, VA length changes may have been overestimated here compared to those occurring in a clinical situation. Furthermore, as the donor temperature was lower than that of a living body, it is possible that biomechanical responses (e.g., flexibility, stiffness) of the soft tissues may have been affected. However, we are unsure how it would be possible to conduct these experiments at, or close to, body temperature and do not believe that this was an important factor during data collection.

Additionally, in patients, the VA experiences not only longitudinal (measured in our study) but also pulsatile circumferential and radial strains due to blood pressure. We made no attempt to pressurize the artery (to mimic radial strains) and did not measure either circumferential or radial strains. Further, no attempt was made to differentiate length changes within the three separate layers of the vessel wall. However, as longitudinal length changes have been implicated primarily as the cause of VA injury, and the current methods likely approximate these length changes, we are confident that we closely represent longitudinal length changes occurring *in-vivo* during CSM [40].

A limitation of the measurement technique is that the piezoelectric crystals measure straight-line inter-crystal distances. As such, if the VA is not straight (as we observed), the inter-crystal distance would be shorter than if the vessel was straight. However, when the VA is not straight, and is slack, then there is no strain or stress imposed on the VA and absolute length and length changes are less important to measure accurately, as ultimately, the interest is in determining when the VA is not slack anymore, becomes a straight line, and becomes strained and experiences longitudinal stress. At that point, which is the crucial part of the VA mechanics, the ultrasound crystals measure accurately the strains from which the associated stresses can be determined accurately.

Another limitation of this study is that VA length changes were measured only for the thrust phase of CSM, no standardized reference length for comparison with literature values of VA length changes occurring at the time of peak force occurrence were available. In other words, a VA length change of 3% measured in this study, could be a length change from 94–97% of the reference length (100%) from a standardized neutral anatomical head and neck configuration [26, 29–31], or it could be from 107 to 110%, or from 123 to 126%. The absence of a standardized reference configuration, thus, does not allow for statements regarding the potential damage of the VA due to over-stretching.

## Conclusions

Head angular displacements and VA length changes were small during CSM thrusts. Of the four CSM procedures measured, mean VA length changes were largest during rotation procedures. This suggests that if clinicians wish to limit VA length changes during the thrust phase of CSM, consideration should be given to the type of CSM used.

## Data Availability

The datasets used and/or analysed during the current study are available from the corresponding author on reasonable request.
